# A Matter of Time: Delayed Presentation and Rapid Progression from Gonadotropin-Independent to Gonadotropin-Dependent Precocious Puberty following Successful Treatment for a Leydig Cell Tumor

**DOI:** 10.1155/2022/5306138

**Published:** 2022-01-06

**Authors:** C. R. Naotunna, D. N. Siriwardana, B. C. Lakmini, M. Samarasinghe, N. Atapattu

**Affiliations:** Lady Ridgeway Hospital, Colombo, Sri Lanka

## Abstract

Leydig cell tumors, most often benign, are a rare cause of isosexual gonadotropin-independent precocious puberty in boys due to secretion of testosterone. Very rarely do these tumors produce estrogen, causing gynecomastia. Testicular sparing surgery is the mainstay of treatment currently although radical orchidectomy was the choice in the past. Following surgery, clinical signs improve along with a revision of biochemical changes. Occasionally, it has been reported few children are progressed to gonadotropin-dependent precocious puberty (GDPP) after initial clinical and biochemical recovery. Gonadotropin receptor analogs have been successful on them to halt the progression of puberty, and growth hormone administration has been used to optimize the adult height. Here, we report a case of a 10-year-old boy who presented very late due to failure in recognition of features of puberty due to a Leydig cell tumor. Even though he underwent successful radical orchidectomy, just within 1 month following surgery, he entered GDPP in contrast to the published cases where it was earliest detected at 3 months.

## 1. Introduction

Leydig cell tumors (LCTs) are a rare cause of gonadotropin-independent precocious puberty in boys [1]. Occasionally, it can progress into gonadotropin-dependent precocious puberty (GDPP) following surgical removal of the mass. We report a case of delayed presentation of LCT with advanced signs of puberty which rapidly progressed to GDPP within a month in contrast to reported cases in the literature [1, 2].

## 2. Case Report

A 10-year-old boy presented with incidental detection of testicular asymmetry by his father complaining the left testes being smaller than the right testes for an unknown duration. The parents did not have any other concerns. Direct questioning revealed that he has had secondary sexual characteristics for a longer period than expected but was assumed to be normal by his parents. He had not encountered any medical professional during this time and had missed the routine school medical inspection at the age of 9 years due to schools being closed following the COVID-19 pandemic. He has been the tallest in the class from preschool. Acne, body hair, and pubic hair have been there for more than 2 years and progressed throughout and deepening of voice for more than 1 year. He has experienced erections for the last 4 months. There were no concerns regarding his behavior.

He had not had any features suggestive of raised intracranial pressure. There was no history of central nervous system infections including tuberculosis, head trauma, or exposure to cranial irradiation.

He denied any abdominal pain or abdominal distension, recently increased skin pigmentation, or salt craving.

His father has achieved puberty at the age of 14 years, and there is no family history of precocious puberty.

**Figure 1:**
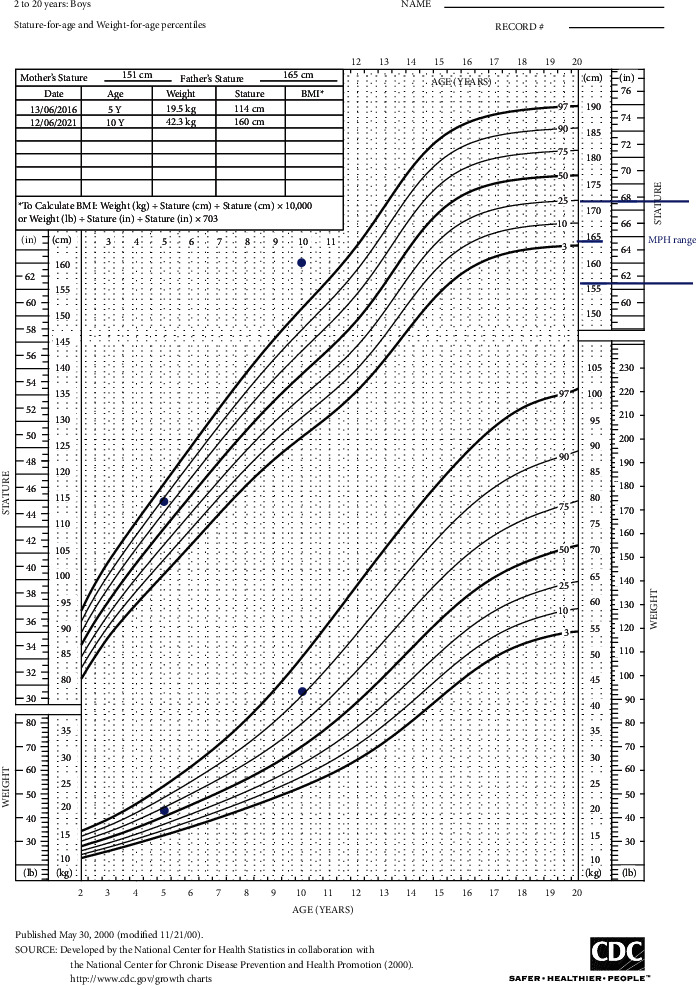
Growth chart of the index case.

On examination, his height was 160.5 cm which was well above the 95th centile and his weight was lying on the 90th centile. His height has exceeded the midparental height range (shown in Figure 1). He was not icteric. He had acnes distributed over the face, increased amount of hair in all 4 limbs, deepened voice, and adult body odor. His puberty assessment was G4 and PH4 with axillary hair and a stretched penile length of 8 cm. His left testis was 3–4 ml. In the right hemiscrotum, there was a hard 10 ml nontender mass. There were no overlying skin changes.

The biochemical evaluation revealed a luteinizing hormone (LH) level of 0.01 IU/L and a follicular-stimulating hormone (FSH) level of 0.05 IU/L, both being suppressed to a greater degree. Beta-HCG, alpha-feto proteins, and dehydroepiandrosterone sulfate (DHEAS) were normal.

Testosterone levels were extremely high (69.45 nmol/L), almost double the upper margin of an adult male reference range (9.7–38.14).

X-ray bone age was 14–15 years according to the Greulich and Pyle method at the chronological age of 10 years.

USS scrotum showed an enlarged right testis with a well-defined heterogeneous hypoechoic lesion measuring 3 × 1.8 cm. Multiple calcifications were seen within the lesion. Contrast-enhanced CT abdomen, pelvis, and scrotum further confirmed a well-localized testicular mass without any evidence of metastasis.

The child underwent a right-sided orchidectomy. Testicular sparing surgery was not possible due to the size of the lesion. Histology revealed a macroscopy of a well-circumscribed solid tumor measuring 30 mm in diameter. On microscopic examination, there was nuclear atypia and bizarre nuclei, but no other features associated with malignant behavior, mitoses <3/10hpf, clear tumor margins, no necrosis, and no vascular invasion. The adjacent seminiferous tubules as well as the distant seminiferous tubules were prepubertal without evidence of spermatogenesis. The immunohistochemistry report was suggestive of a Leydig cell tumor.

1 month following surgery, even though the serum testosterone level has come down to 12.69 nmol/l, hormonal assay revealed that the child has now entered gonadotropin-dependent puberty with an LH value of 10.61 IU/L and FSH 9.59 IU/L.

As the bone age was already advanced, it was decided not to proceed with GnRH analogs.

## 3. Discussion

A Leydig cell tumor (LCT) is a sex cord-stromal tumor and accounts for 1–3% of all testicular tumors in adults and 4–9% in prepubertal children [2]. Even though it can occur at any age, among children, it is most commonly present in the prepubertal age between 5 and 10 years and is benign [3]. Most LCTs are unilateral and palpable or detected in an ultrasound scan (USS), and only 3–10% will have bilateral involvement [4]. An LCT produces androgens, namely, testosterone, androstenedione, dehydroepiandrosterone sulfate, and 17*α*-hydroxyprogesterone, and sometimes estrogen either by peripheral aromatization of testosterone or else directly produce estradiol. When androgens are the predominant secreting hormone, boys present with features of gonadotropin-independent precocious puberty, penile enlargement, pubic hair growth, masculinization, advanced bone age, and voice changes. Boys who have an estrogen-producing LCT will present with gynecomastia and breast tenderness [3].

Biochemically, LCT will show elevated serum testosterone levels and increased estradiol levels if estrogen is secreted, along with prepubertal levels of FSH and LH. In pure LCT alpha-feto proteins, beta-HCG levels would be typically normal [3]. USS of the scrotum will reveal a homogenous hypoechoic appearance.

The histology and the immunohistochemistry are important aspects, deciding the future management and treatment modalities. In contrast to the index case where the adjacent nonneoplastic testicular tissue was prepubertal, there are reported cases where the adjacent seminiferous tubules were positive for spermatogenesis with distant infantile seminiferous tubules [5, 6].

Orchidectomy is the mainstay of therapy with an overall good prognosis and regression of clinical signs though recently testicular sparing surgery has become a choice of treatment [1, 4].

In most instances, following treatment, the signs of puberty have regressed, but occasionally, following surgery, some have entered gonadotropin-dependent precocious puberty (GDPP). A review conducted in 2015 reported 8 cases [1], two cases in 2018 [4], and only one case 2020 [2] presenting with GDPP following surgery. GDPP was detected as early as 3 months after surgery, and in some cases, it was detected as late as 1 year after surgery in contrast to our case where GDPP was detected just 1 month after surgery.

In all these reported cases, patients have presented much earlier and received gonadotropin-releasing analog (GnRH analogs) except for the oldest patient who was 9 years at the presentation with a bone age of 13.5 years. GnRH analogs have successfully withheld the progression of puberty, but the already advanced bone age resulted in growth deceleration, and growth hormone has been administered to some patients to optimize the adult height [4]. The exact mechanism of what leads to GDPP is not clear but believed to be due to early maturation on GnRH pulse generator following exposure to the high concentration of testosterone [1].

The long-term follow-up in adult males with LCT has found low sperm counts and abnormal sperm morphology with low testicular volume and has been a known cause for male infertility [7].

## 4. Conclusions and Learning Point

It is important to recognize features of precocious puberty by caregivers to prevent delaying presentation and diagnosis of sinister conditions. Gonadotropin-dependent puberty should be considered when clinical signs failed to regress following surgery, and therefore, these children require short-term as well as long-term follow-up.

## Data Availability

The data supporting the findings of this case are available within the article.
